# Potential Use of Pharmacogenetics to Reduce Drug-Induced Syndrome of Inappropriate Antidiuretic Hormone (SIADH)

**DOI:** 10.3390/jpm11090853

**Published:** 2021-08-28

**Authors:** Russell A. Wilke

**Affiliations:** Sanford School of Medicine, University of South Dakota, 1400 West 22nd Street, Sioux Falls, SD 57105, USA; russell.wilke@usd.edu

**Keywords:** neurohypophysis, antidiuretic hormone, adverse drug reaction, drug safety

## Abstract

Syndrome of inappropriate antidiuretic hormone (SIADH) is a common cause of hyponatremia, and many cases represent adverse reactions to drugs that alter ion channel conductance within the peptidergic nerve terminals of the posterior pituitary. The frequency of drug-induced SIADH increases with age; as many as 20% of patients residing in nursing homes have serum sodium levels below 135 mEq/L. Mild hyponatremia is associated with cognitive changes, gait instability, and falls. Severe hyponatremia is associated with cerebral edema, seizures, permanent disability, and/or death. Although pharmacogenetic tests are now being deployed for some drugs capable of causing SIADH (e.g., antidepressants, antipsychotics, and opioid analgesics), the implementation of these tests has been based upon the prior known association of these drugs with other serious adverse drug reactions (e.g., electrocardiographic abnormalities). Work is needed in large observational cohorts to quantify the strength of association between pharmacogene variants and drug-induced SIADH so that decision support can be developed to identify patients at high risk.

## 1. Background

Clinical derangements in salt and water homeostasis are common [1,2]. Hyponatremia (low serum sodium level) is the most common electrolyte abnormality seen in routine practice [3,4]. Mild hyponatremia (serum sodium < 135 mEq/L) can lead to cognitive changes, gait instability, and falls. Severe hyponatremia (serum sodium < 125 mEq/L) causes profound osmotic disturbances and fluid shifts in multiple organ systems. If left uncorrected, the accompanying fluid shifts lead to cerebral edema, seizures, disability, and/or death [5]. The societal cost of this problem is enormous [6,7].

Age is a strong predictor of hyponatremia. Nearly one in five patients residing in nursing homes have serum sodium levels below 135 mEq/L, and this patient population is at risk of serious injury if they fall [8]. In a series of 696 emergency room patients with advanced age (mean age 86.1 ± 5.6 years), the prevalence of mild hyponatremia was 26% in patients with falls (95% CI: 19.8–32.4), and 13% in patients without falls (95% CI: 10.1–16.3) [9]. In the inpatient setting, the prevalence of mild hyponatremia is 6% on general medical wards (95% CI 5.9–6.1), and 22% on geriatric wards (95% CI 20.2–24.3) [8]. Severe hyponatremia occurs at a rate of 1% on general wards (95% CI 0.7–0.8), and 4% on geriatric wards (95% CI 3.0–6.1). When analyses are restricted to cases of very severe hyponatremia (serum sodium < 116 mEq/L), the large majority of cases are drug-induced [10].

## 2. Excessive Antidiuretic Hormone

Hyponatremia can be caused by a variety of mechanisms. Clinical assessment begins with an assessment of volume status [3]. While hyponatremia can accompany volume contraction (e.g., renal salt wasting in dehydration) [11] or volume overload (e.g., uncompensated heart failure) [12], most cases occur in patients that are relatively euvolemic [1]. The presence of low serum sodium levels in a patient with normal volume status suggests the presence of a neuroendocrine abnormality such as hypothyroidism, adrenal insufficiency, or excessive amounts of circulating antidiuretic hormone (ADH).

**Figure 1:**
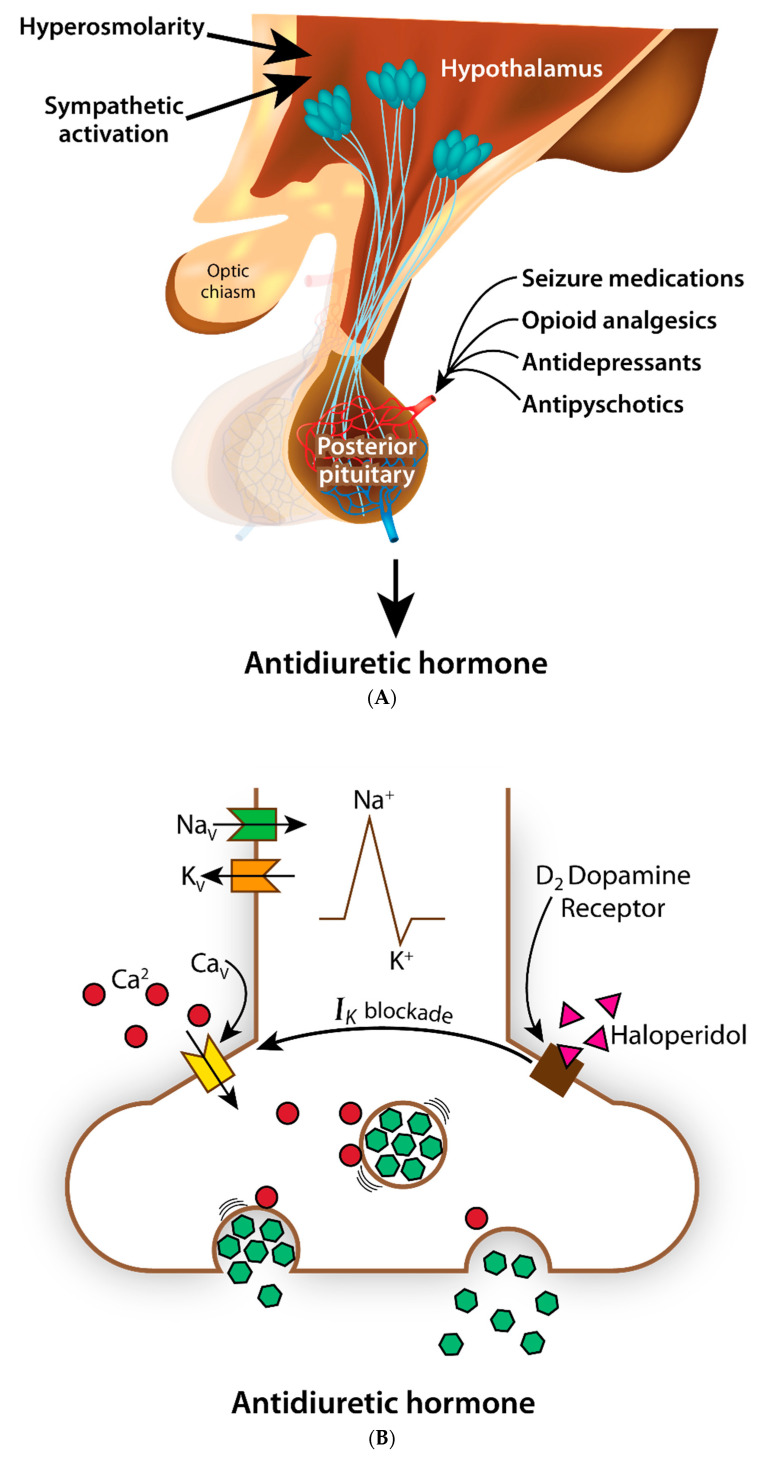
(**A**) SIADH as an adverse drug reaction; (**B**) Peptidergic nerve terminals release ADH.

Antidiuretic hormone, also called vasopressin, is a 9-amino acid peptide with a complex physiological role that includes the regulation of renal free water excretion and the maintenance of vascular tone [13]. Circulating ADH is primarily derived from nerve terminals of the posterior pituitary gland (the neurohypophysis). These peptidergic nerve terminals represent distal extensions of axons originating in the hypothalamus. They are structurally and functionally distinct from the glandular tissue of the anterior pituitary gland (the adenohypophysis). When serum sodium levels rise above 145 mEq/L, hypothalamic osmoreceptors located in the supraoptic and paraventricular nuclei activate secretion of ADH directly from the posterior pituitary [14] (Figure 1A). The resulting increase in systemic levels of ADH then leads to enhanced water reabsorption by the kidneys. The binding of ADH to its receptors on the basolateral membrane of the renal collecting tubules leads to the insertion of aquaporin-2 water channels within the apical membrane, thereby facilitating the maintenance of osmotic homeostasis through the retention of free water. It is generally accepted that this finely tuned physiological process contributed to the survival of land-dwelling organisms during evolution.

While hypothalamic control of ADH release from the posterior pituitary is tightly regulated, this process is also highly sensitive to perturbation by a variety of endogenous and exogenous insults. Excessive ADH levels are commonly seen in patients with brain trauma [15], as well as in patients with space-occupying lesions (e.g., lymphoma) [16] or infections located in the base of the brain (e.g., meningitis) [17]. The link between infection and posterior pituitary dysregulation is not, however, restricted to infections of the central nervous system. Low serum sodium levels occur in as many as one in three patients with pneumonia, indicating that excessive ADH levels can accompany systemic infections as well [2,4]. During states of severe inflammation, cytokine-induced changes in posterior pituitary signal transduction tend to drive circulating levels of ADH upward with the undesirable effect of driving serum sodium levels downward. Within this context, hyponatremia is a strong predictor of mortality risk. In patients hospitalized with COVID-19, for example, each 1 mEq/L drop in serum sodium level is associated with a 14% increase in the risk of death [4].

Thus, elevated levels of ADH can be adaptive (e.g., to maintain blood volume during states of poor cardiac output such as congestive heart failure) or maladaptive (e.g., during states of severe inflammation). Maladaptive elevation in circulating ADH level—causing an inappropriately high urine osmolality and decreased serum sodium level in a patient with adequate blood volume—is frequently referred to as the “syndrome of inappropriate antidiuretic hormone (SIADH).” This term was coined nearly half a century ago [18]. It is a fairly nonspecific term, and as noted above, the presence of “SIADH” can simply reflect subtle changes in posterior pituitary function accompanying trauma, tumor, or infection. More often, however, excessive ADH levels reflect an adverse reaction to a drug.

## 3. SIADH as an Adverse Drug Reaction

Several classes of commonly used drugs are capable of causing SIADH through mechanisms involving the central nervous system. Many antidepressants (e.g., selective serotonin reuptake inhibitors, SSRIs) lead to increased release of ADH from the posterior pituitary by potentiating the activation of adrenergic receptors within the hypothalamus (Figure 1A). Data from the FDA Adverse Event Reporting System (AERS) have recently been leveraged to quantify the relationship between hyponatremia and antidepressant use based upon binding affinities for individual drugs to adrenergic receptor subtypes and serotonergic receptor subtypes, as well as to serotonin transporters, dopamine transporters, and norepinephrine transporters [19]. Using a linear regression model, the final adjusted reporting odds ratio for association between hyponatremia and antidepressant use was 1.91 (95% confidence interval 1.83–2.00) [19]. A significant linear correlation was found for hyponatremia and binding affinity for the adrenergic receptors. The association was strongest for mirtazapine, followed by the SSRIs.

It has long been known that SSRIs can cause drug-induced SIADH. Hyponatremia occurs in as many as one in three patients taking an SSRI [20]. Many antipsychotic agents (including both typical and atypical dopamine receptor antagonists) also cause drug-induced SIADH [21], in part by attenuating K^+^ conductance within posterior pituitary nerve terminals [22]. An example is provided schematically in Figure 1B, wherein dopaminergic antagonists inadvertently alter ion channel function leading to excessive ADH release as an adverse drug reaction. Similar perturbations in posterior pituitary membrane conductance have been observed for opioid analgesics [23,24,25]. In clinical practice, however, the strength of association between opioids and SIADH does not appear to approach the magnitude of the association between SSRIs and SIADH [26]. Another class of drugs strongly associated with SIADH has been the anti-seizure drugs [27]. Carbamazepine and oxcarbazepine cause hyponatremia quite often [28]. As many as one in four patients using carbamazepine in a seizure clinic develop hyponatremia [29]. Like many of the drugs discussed above, these agents also increase circulating ADH levels through a central mechanism involving the regulation of membrane bound ion channels [30].

## 4. Importance of Establishing Mechanism

Mechanism is a key consideration when considering a diagnosis of drug-induced SIADH. Central causes of hyponatremia must be distinguished from peripheral causes. For example, nonsteroidal anti-inflammatory drugs (NSAIDs) such as naproxen can cause hyponatremia peripherally by potentiating the effects of ADH at the level of the kidney (altering prostaglandin levels in the loop of Henle and renal collecting ducts) [31]. This is not SIADH. Other agents, such as antineoplastic agents, are capable of causing hyponatremia through mechanisms that are both central and peripheral [32,33]. In order to accurately diagnose drug-induced SIADH, three conditions must be met. (1) There needs to be clear documentation of hypotonic hyponatremia caused by a disruption in pituitary homeostasis (i.e., retention of excess free water in the general circulation). (2) Paraneoplastic processes need to be ruled out, because some neuroendocrine tumors are capable of producing ADH ectopically [34]. (3) There needs to be a strong temporal relationship linking the hyponatremia with administration of a drug capable of altering posterior pituitary function [35].

“Naranjo’s algorithm” is frequently used to assess causality for a variety of adverse drug reactions, based on temporal relationships (onset and resolution) in longitudinal datasets [20]. When already available, data regarding re-challenge are particularly helpful. However, given the high potential for morbidity and mortality accompanying drug-induced hyponatremia, intentional re-challenge with a suspect drug should typically be avoided [36]. In general, the application of Naranjo’s algorithm has been very effective at confirming drug-induced SIADH. In a cohort of 198 patients who had SIADH and a prior documented exposure to at least one suspect drug, nearly 75% of these cases were confirmed as drug-induced using Naranjo’s algorithm [35]. This approach has been particularly effective at identifying cases of SIADH caused by SSRIs [20,35].

Once a diagnosis is confirmed, treatment of drug-induced SIADH varies according to the severity of the hyponatremia [37]. Many patients can simply be managed by removal of the offending agent and restriction of oral water intake. In some patients, clinicians also choose to attenuate the central secretion of ADH with demeclocycline [38] or optimize renal mobilization of free water with emerging agents such as SGLT-2 inhibitors [39]. Urea, loop diuretics, and selective ADH/vasopressin-receptor antagonists can also be used to increase free water excretion [1,17]. Conivaptan is an ADH/vasopressin antagonist available in an intravenous formulation for the treatment of severe SIADH. Tolvaptan is an oral formulation approved for use in patients with SIADH or hyponatremia due to congestive heart failure [40].

## 5. The Need for Risk Stratification

In summary, drug-induced SIADH is a common adverse drug reaction with potentially life-threatening consequences. It would therefore be advantageous to identify which patients are at highest risk prior to exposing them to drugs capable of altering neurohypophysial physiology. Although the field of pharmacogenetics has made great strides in assessing markers of risk for many clinically severe adverse drug reactions, the genetic architecture underlying drug-induced SIADH remains relatively uncharacterized. Large observational cohorts are now positioning themselves to address these and other similar questions retrospectively [41], and multi-institutional consortia are creating robust infrastructure to quantify the impact of gene-based dosing prospectively through pragmatic clinical trials involving many of these drugs [42].

In general, serum sodium level is a heritable trait. For baseline serum sodium levels, heritability estimates in twin studies have ranged from 0.41 (95% confidence interval: 0.35–0.46) to 0.49 (95% confidence interval: 0.43–0.54) [43]. This means that nearly half of all variance in serum sodium levels may be attributable to genetic factors. Furthermore, early genome-wide association studies (GWAS) have also identified a small number of loci associated with serum sodium level in cohorts of unrelated individuals. *NFAT5*, for example, encodes a transcription factor that modulates intracellular response to hypertonic stress, and variants at this locus are associated with serum sodium level in individuals of varying ancestry [44]. Less is known, however, about genetic loci linked to *change* in serum sodium level over time (i.e., in longitudinal data rather than cross-sectional data). Some work has been conducted to identify loci impacting peripheral (renal) changes in salt and water homeostasis associated with diuretic use [45], but very little is known about the genetic determinants of centrally mediated changes in salt and water homeostasis including the changes seen in drug-induced SIADH.

Genes shown to influence drug response are typically categorized as either pharmacodynamic genes (influencing a drug’s mechanism of action) or pharmacokinetic genes (influencing a drug’s absorption, distribution, metabolism, or elimination) [46]. For drug-induced SIADH, genes influencing mechanism of action would include receptors, G-proteins, and ion-channels expressed in the hypothalamus and the posterior pituitary (Figure 1A,B). While there is great variability in the genes known to encode many relevant receptors, transporters, and ion channels [47], very little work has been performed to quantify the impact of these variants on ADH release. There is evidence, however, to indicate that genetic variability within G-proteins can lead to SIADH. Variants in the stimulatory alpha subunit (Gsα) known to regulate intracellular cyclic AMP levels have been reported in a patient with a neonatal form of SIADH [48].

Pharmacokinetic genes are also likely to increase the risk for drug-induced SIADH. Clearly, variability in pharmacokinetic genes can lead to serious adverse outcomes for many of the drugs discussed in this review. Cytochrome P450 (CYP) 2C19, for example, metabolizes 5–10% of all prescription drugs, and loss of function alleles at the *CYP2C19* gene locus are known to increase risk for potentially lethal ventricular arrhythmias in patients using three commonly prescribed SSRIs: sertraline, citalopram, and escitalopram [49,50]. Furthermore, loss of function alleles at the *CYP2D6* locus increase patient risk for ventricular arrhythmias in the context of three other SSRIs: fluoxetine, fluvoxamine, and paroxetine [49,51]. Similar associations exist for *CYP2D6* variants and therapeutic failure in the context of opioids [52,53], and variants in a third candidate cytochrome P450 gene, *CYP2C9*, have also been linked to altered outcomes for anti-seizure drugs [54,55].

Pharmacogenetic tests are already being deployed clinically to guide prescribing for many drugs discussed in this review. However, the implementation of these tests is currently based on known association with severe adverse drug reactions other than SIADH. For example, decision support already exists within electronic medical records at some academic institutions to optimize the efficacy of SSRIs and reduce the frequency of adverse events such as electrocardiographic abnormalities associated with their use [50,56]. It is highly likely that the same gene-based dosing approach would reduce the frequency of SIADH associated with the use of SSRIs. Because drug-induced SIADH is associated with significant morbidity (cognitive changes, gait instability and falls) and mortality (cerebral edema, seizures, and death), the genetic architecture underlying this common adverse drug reaction needs to be defined in multi-institutional cohorts.
